# Functional examination of novel kisspeptin phosphinic peptides

**DOI:** 10.1371/journal.pone.0195089

**Published:** 2018-04-03

**Authors:** Xiaoyang Zhang, Magdalini Matziari, Yixin Xie, David Fernig, Rong Rong, Jia Meng, Zhi-Liang Lu

**Affiliations:** 1 Department of Biological Sciences, Xi’an Jiaotong-Liverpool University, Suzhou, Jiangsu, China; 2 Department of Chemistry, Xi’an Jiaotong-Liverpool University, Suzhou, Jiangsu, China; 3 Department of Biochemistry, Institute of Integrative Biology, University of Liverpool, Liverpool, United Kingdom; University of South Alabama Mitchell Cancer Institute, UNITED STATES

## Abstract

Kisspeptins acting on their cognate G protein-coupled receptor, kisspeptin receptor, play important roles in the suppression of cancer cell metastasis and regulation of the reproductive system, and therefore are important for therapeutic intervention. All native functional human kisspeptins (kisspeptin-54, kisspsptin-14 and kisspeptin-13) share the 10 amino acids of kisspeptin-10 at their C-terminus (45–54). However, they are inactivated rapidly by matrix metalloproteinases (MMPs) through the cleavage of the peptide bond between glycine^51^ and leucine^52^, which limits their clinical applications. Development of MMP-resistant analogues of kisspeptins may provide better therapeutic outputs. In the present study, two kisspeptin phosphinic peptides were designed and synthesized, and their ability to induce phosphorylation of ERK1/2 through kisspeptin receptor and their inhibition on MMP-2 and MMP-9 whose activity correlates with cancer metastasis were assessed. The results showed that one analogue, phosphinic kisspeptin R isomer (PKPR), exhibited kisspeptin receptor-agonistic activity and also inhibitory activity on MMP-2, indicating that PKPR may serve as a lead for the further development of kisspeptin analogues for therapeutic purpose.

## Introduction

Kisspeptin receptor, that is previously known as GPR54 [1], AXOR12 [2] and hOT7T175 [3], is a member of G protein-coupled receptor (GPCR) superfamily and is coupled to G_q/11_ proteins [4]. Its endogenous ligands are firstly isolated from human placental extracts and termed either metastin (54-amino acid peptide) due to its capability to inhibit cancer metastasis [3] or kisspeptins (KPs) with different length in amino acids (54-, 14- and 13-amino acid peptides) [5]. In humans, KP-54 is cleaved from a 145-amino acid polypeptide precursor encoded by *KiSS1* gene [3]. KP-54 is further hydrolysed into several shorter and biologically functional variants, i.e. KP-14, KP-13 and KP-10. The different forms of KPs possess similar receptor binding affinity and potency [3] and share the 10 amino acids of KP-10 at their C-terminus, which are highly conserved among all vertebrate species [6].

KPs and their cognate receptor are key regulators of the mammalian reproductive system. Natural mutations of the human kisspeptin receptor lead to idiopathic hypogonadotropic hypogonadism [7–10]. Administration of KPs stimulates the release of both luteinizing hormone (LH) and follicle-stimulating hormone in mammalian species, including humans [11, 12], mice [13, 14], rats [15] and also many domestic species such as cattle and sheep [16]. Detailed studies in the past reveal that KP-induced release of LH is inhibited by GnRH receptor antagonists, indicating that GnRH is the mediator of KP-induced release of gonadotropins [15]. The expression of kisspeptin receptor at mRNA [14, 15] and protein levels [13] is also observed in the GnRH neurons. These results find that KPs regulate the reproductive system by activating their receptor presented on the GnRH neurons, which leads to the secretion of GnRH and, consequently, the release of gonadotropins.

Originally, the *KiSS1* gene is identified as a metastasis suppresser gene due to the ability of KP-54 to inhibit cancer cell metastasis [17]. KPs inhibit metastasis in a variety of cancers, including melanoma [3], breast [18], endometrial [19], gastric [20] ovarian cancers [21], and others. Therefore, KP analogues may act as inhibitors of cancer metastasis which is the major cause of cancer death.

However, native KPs are metabolically unstable in blood and are hydrolysed by various serum-containing proteases and MMPs such as MMP-2 and MMP-9 [22, 23]. MMPs cleave the peptide bond between Gly^51^-Leu^52^ of KPs [24]. The expression level of MMP-2 and MMP-9 increases during the progression of many cancers, such as breast [25, 26], endometrial [27, 28] and ovarian [29, 30] cancers, which closely correlates with cancer migration and poor prognosis. Therefore, development of KP analogues which activate kisspeptin receptor and resist or inhibit MMPs may provide a novel treatment to inhibit cancer cell metastasis or for hormone replacement therapies with enhanced potency. Two main approaches are used to improve metabolic stability of KPs, including reduction of peptide length of KPs [31] and/or substitutions of the cleavage sites with nonhydrolysable isosteres [23, 32]. In the present study, two penta-peptides are designed and synthesized based on the primary structure of KP-10 among which the last C-terminal 6 amino acids are crucial for receptor binding [32]. To develop KP agonistic analogues which bind and inhibit MMPs, the peptide bond between Gly-Leu of the C-terminal 6 amino acid peptide is replaced by a phosphinic acid moiety, -PO_2_-CH_2_-. The synthetic peptides containing substitution of a peptide bond with the phosphinic acid moiety are termed KP phosphinic peptides. Phosphinic peptides are potent and selective inhibitors of various proteases, such as MMPs [33, 34], as their chemical structure mimics the intermediate transition state formed during the hydrolysis of peptides by proteases. In the present study, the kisspeptin receptor-agonistic activity and the inhibitory activity on MMP-2 and MMP-9 of the synthetic peptides are examined, finding that one synthetic peptide analogue activates kisspeptin receptor and inhibits MMP-2 activity.

## Materials and methods

### Materials

The cDNA of flag-tagged kisspeptin receptor was kindly provided by Dr. Andy Babwah, the University of Western Ontario, Canada. KP-10 and Sulfo-Cy^5^-KP-18 were commercially synthesized by Chinese Peptides (Hangzhou, China) to a purity >95%. A water soluble Sulfo-Cy^5^-NHS was used to conjugate with the N-terminal amine of KP-18. Rabbit monoclonal anti-ERK1/2 and mouse monoclonal anti-β-actin antibodies were purchased from Cell Signalling Technology (Boston, USA). IRDye® goat anti-mouse or anti-rabbit IgG (H + L) secondary antibody was from LI-COR (Lincoln, USA). Fetal bovine serum (FBS) was purchased from Bovogen (East Keilor, Australia) and all other reagents used in cell culture were purchased from Gibco (Waltham, USA). The MMP colorimetric drug discovery kits were purchased from Enzo Life Sciences (New York, USA). All other reagents were purchased from Sigma-Aldrich (St. Louis, USA) and Thermofisher Scientific (Waltham, USA).

### Synthesis and purification of KP phosphinic peptides

**Fig 1:**
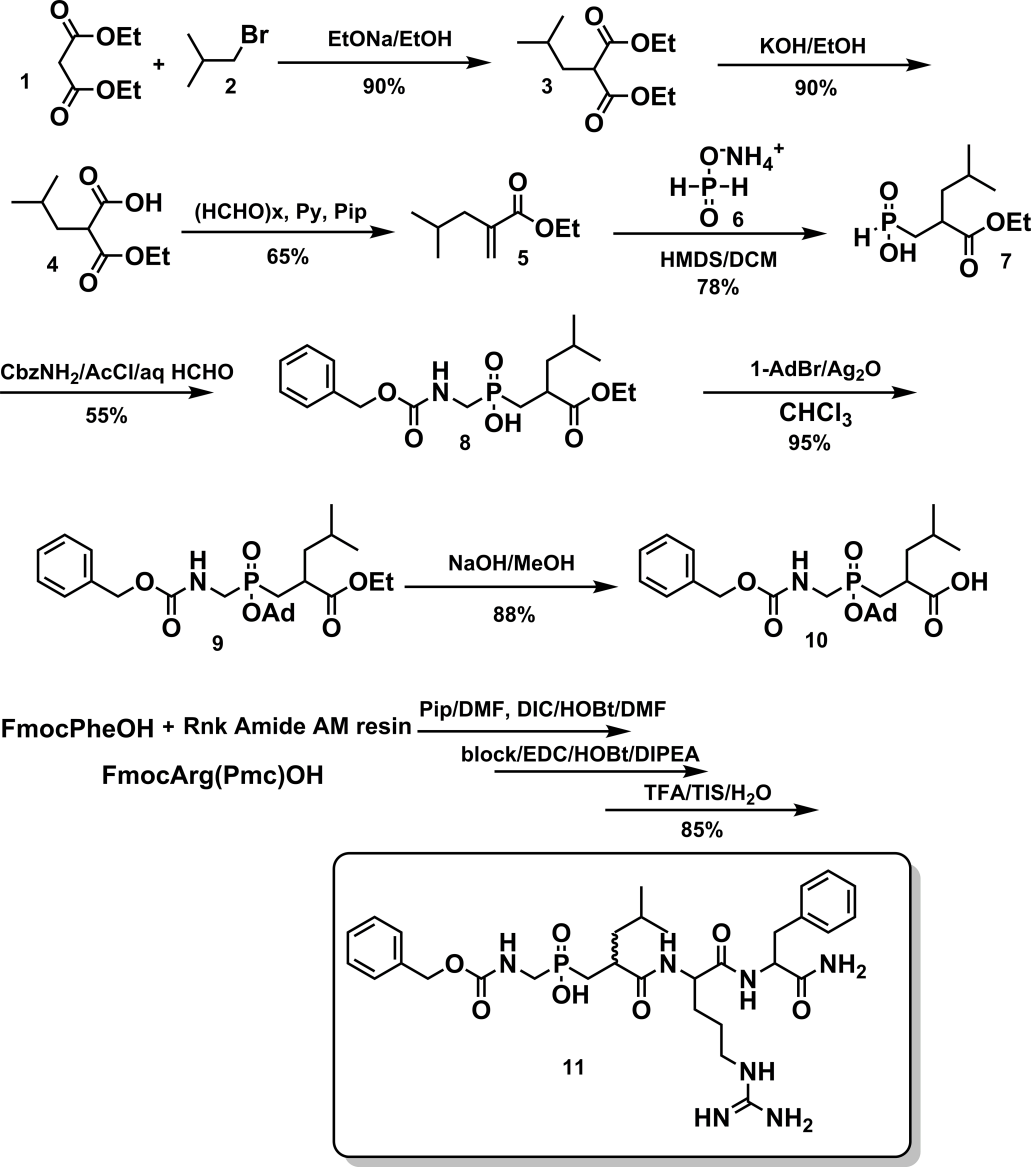
Synthesis of the KP phosphinic peptides.

The applied synthetic procedure is depicted in Fig 1. The phosphinic pseudodipeptide block **10** was synthesized by a 3-component condensation [35] in order to avoid the cumbersome and low-yielding synthesis of the aminophosphinic Gly analogue. In detail, the acrylate precursor of Leu **5** was synthesized by alkylation of the malonate ester **1** that led to **3**, selective monosaponification produced **4** which upon Knoevenagel condensation with formaldehyde led to **5** [36]. The synthesis of **7** was accomplished by a Michael-type addition of hypophosphorus ammonium salt **6** under inert atmosphere using HMDS as the silylation agent. The phosphinic acid **7** was subsequently condensed with formaldehyde and Cbz-carbamate in AcCl that acts as both solvent and acetylation reagent which facilitates the intermediate imine formation. The phosphinate pseudodipeptidic block **8** was afterwards protected at the phosphinate with the adamantyl group towards **9** [37], which was then saponified to lead to compound **10**. This block was used in solid-phase peptide synthesis by using Rink-amide lanterns and standard Fmoc-protocol. The 2 isomers of **11** were further separated by RP-HPLC. All intermediates and the final products were identified and characterized by HRMS and NMR. The assignment of the absolute configuration was based on previous studies of similar phosphinic peptide structures [38].

### Cell culture and receptor transfection

HEK293 (ECACC 85120602) and MCF-7 (ECACC 86012803) cells were cultured in Dulbecco’s modified Eagle’s medium (DMEM) supplemented with 10% (v/v) FBS, 2 nM L-glutamine, 0.2 unit/ml penicillin, 0.2 μg/ml streptomycin and were maintained at 37°C in a humidified atmosphere containing 5% (v/v) CO_2_. Both cell lines were reported to express kisspeptin receptor naturally [39, 40], but no receptor binding and activity were detected in our cells. HEK293 and MCF-7 cells were transiently transfected with 10 μg cDNA of human kisspeptin receptor by electroporation using the Bio-Rad Gene Pulser Xcell™ system and Bio-Rad 0.4 cm electroporation cuvettes. HEK293 cells were electroporated at 300 volts with a capacitance of 950 μF, while MCF-7 cells were electroporated at 320 volts with a capacitance of 950 μF.

### Western blot analysis of the phosphorylation of ERK1/2

HEK293 and MCF-7 cells transiently transfected with kisspeptin receptor were used for phosphorylation assay of the extracellular signal-regulated kinase 1/2 (ERK1/2) After 24 hours following transfection, the cells were serum-starved in a serum-free medium (DMEM supplemented with 10 mM 4-(2-hydroxyethyl)-1-piperazineethanesulfonic acid (HEPES), 0.1% (w/v) bovine serum albumin (BSA), 2 nM L-glutamine, 0.2 unit/ml penicillin and 0.2 μg/ml streptomycin) overnight prior to any treatment. Assays were then conducted by stimulating the cells with KP-10 or KP phosphinic peptides. Following stimulation, the cells were placed on ice and solubilized with SDS-PAGE sample loading buffer supplemented with 5 mM sodium orthovanadate and complete^TM^ protease inhibitor cocktail. Solubilized samples were boiled and then subjected to SDS-PAGE. Subsequently, proteins were electrotransferred onto polyvinylidene difluoride (PVDF) membranes for immunoblotting. Immunoblots were then assayed for phosphorylated ERK1/2 using a phospho-specific rabbit monoclonal antibody (1:1000) (phospho-p42/44 ERKs, Thr^202^/Tyr^204^) and for β-actin using a mouse monoclonal antibody (1:1000). Following incubation with IRDye® goat anti-mouse (1:5000) or anti-rabbit (1:10000) IgG (H + L) secondary antibody, the PVDF membranes were visualised using Odyssey® infrared imaging system (LI-COR, Lincoln, USA). Immunoblots were quantified by densitomery using the Odyssey® application software (version 3.0).

### Evaluation of receptor binding with Sulfo-Cy^5^-KP-18

HEK293 cells transiently transfected with kisspeptin receptor were seeded onto 10-cm cell culture dishes. After 24 hour-incubation in complete medium, the cells were washed twice with phosphate-buffered saline (PBS), trypsinised and seeded onto 96-well cell culture plates (8 x 10^4^ cells/well). On the following day, the cells were washed once with PBS and then incubated with either vehicle (0.02% (v/v) propylene glycol) or various concentrations of Sulfo-Cy^5^-KP-18 in the serum-free DMEM containing 10 mM HEPES and 0.1% BSA for 4 hours at 4°C. The non-specific binding was determined in the presence of 10 μM KP-10. After incubation, free ligands were removed by three rapid washes with ice-cold PBS. The fluorescence intensity was measured using PHERAStar FS microplate reader (BMG LABTECH, Germany).

### Sulfo-Cy^5^-KP-18-induced internalisation of kisspeptin receptor

MCF-7 cells transiently transfected with kisspeptin receptor were seeded onto 96-well plates. After 48 hours, the cells were incubated with a mixture of 50 nM Sulfo-Cy^5^-KP-18 and various concentrations of tested peptides or their corresponding vehicles in the complete medium respectively for 30 minutes at 37°C. After incubation, free ligands were removed by rapid washes with ice-cold PBS and the non-internalized ligands were removed by a wash with ice-cold 0.1 M glycine-HCl buffer (pH 3.0) at 4°C for 5 minutes. The fluorescence intensity was measured using PHERAStar FS microplate reader.

### Measurement of the inhibition of KP phosphinic peptides on the activity of MMPs

The activity of MMPs was measured using MMP colorimetric drug discovery kits in a 96-well microtiter plate according to the manufacturer’s instructions. Different concentrations of the phosphinic peptides or 1.3 μM N-Isobutyl-N-(4-methoxyphenylsulfonyl)glycyl hydroxamic acid (NNGH), a positive inhibitor for MMPs, were, respectively, added to the assay mixtures containing MMP enzyme (MMP-2 or MMP-9; 9 mU/μL). The plates were then incubated at 37°C for 1 hour for enzyme reaction. After that, a final concentration of 100 μM colorimetric substrate (Ac-PLG-[2-mercapto-4-methyl-pentanoyl]-LGOC2H5) was added to each well and the plates were then read at 412 nm every minute for at least 20 minutes using PHERAStar FS microplate reader. The full enzyme activity (control) was measured without addition of any inhibitor or tested peptides. The reaction velocity (V) was determined by calculating the slope of the fitted line to the linear portion of the data collected. The slope of blank (without addition of MMP enzyme) was subtracted from all samples and the remaining activity of the enzyme was calculated using following formula.

$$\%\;Enzyme\;activity=\frac{V(inhibitor)}{V(control)}*100$$

### Data analysis

The means ± SD are shown for values obtained for the numbers of independent experiments indicated in the figure legends. GraphPad Prism software 6.0 (Graph Pad, San Diego, USA) was used to analyse the data for statistical significance. The statistical significance was calculated by one-way or two-way ANOVA.

## Results

### Design and synthesis of KP phosphinic peptides

**Fig 2:**
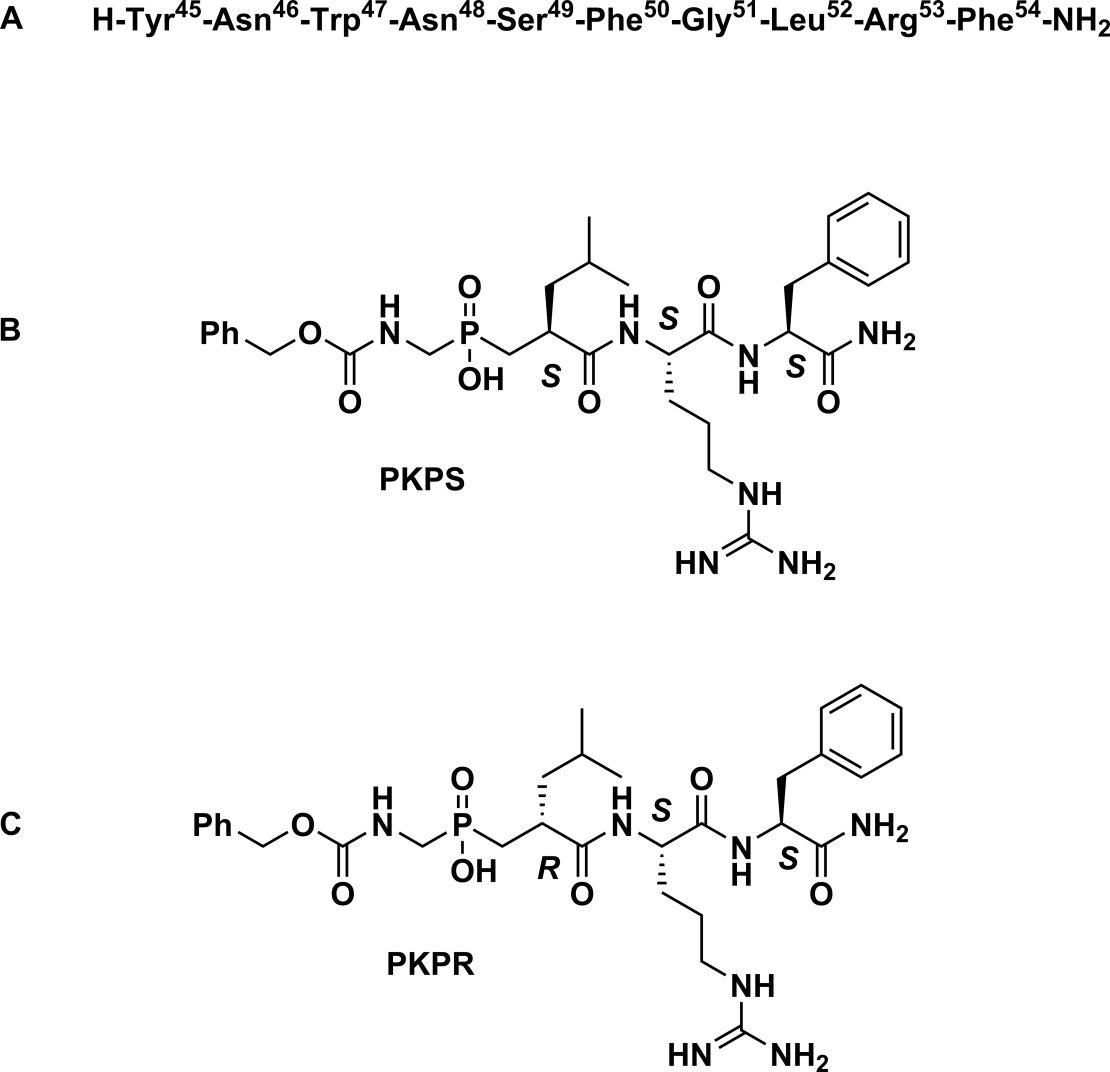
Structures of KP-10 and its derived phosphinic peptides. The amino acid sequence of KP-10 is shown in A). The predicted structures of phosphinic kisspeptin S isomer (PKPS) and phosphinic kisspeptin R isomer (PKPR), which are diastereoisomers, are shown in B) and C).

The KP phosphinic peptides were designed from the C-terminal 5 amino acids of KP-10 by replacement of the amide bond between Gly^51^ and Leu^52^ with the phosphinate (-P(O)(OH)CH_2_) bond which is resistant to hydrolysis of MMPs (Fig 2). The N-terminus of the KP phosphinic peptides was protected by the Cbz group which resembles the Phe^50^ side-chain of KP-10. The synthesis is described in section 2.2. The KP phosphinic peptides have S isomer (PKPS) and R isomer (PKPR) which are diastereoisomers (Fig 2).

### Effect of KP phosphinic peptides on kisspeptin receptor-elicited ERK1/2 phosphorylation

**Fig 3:**
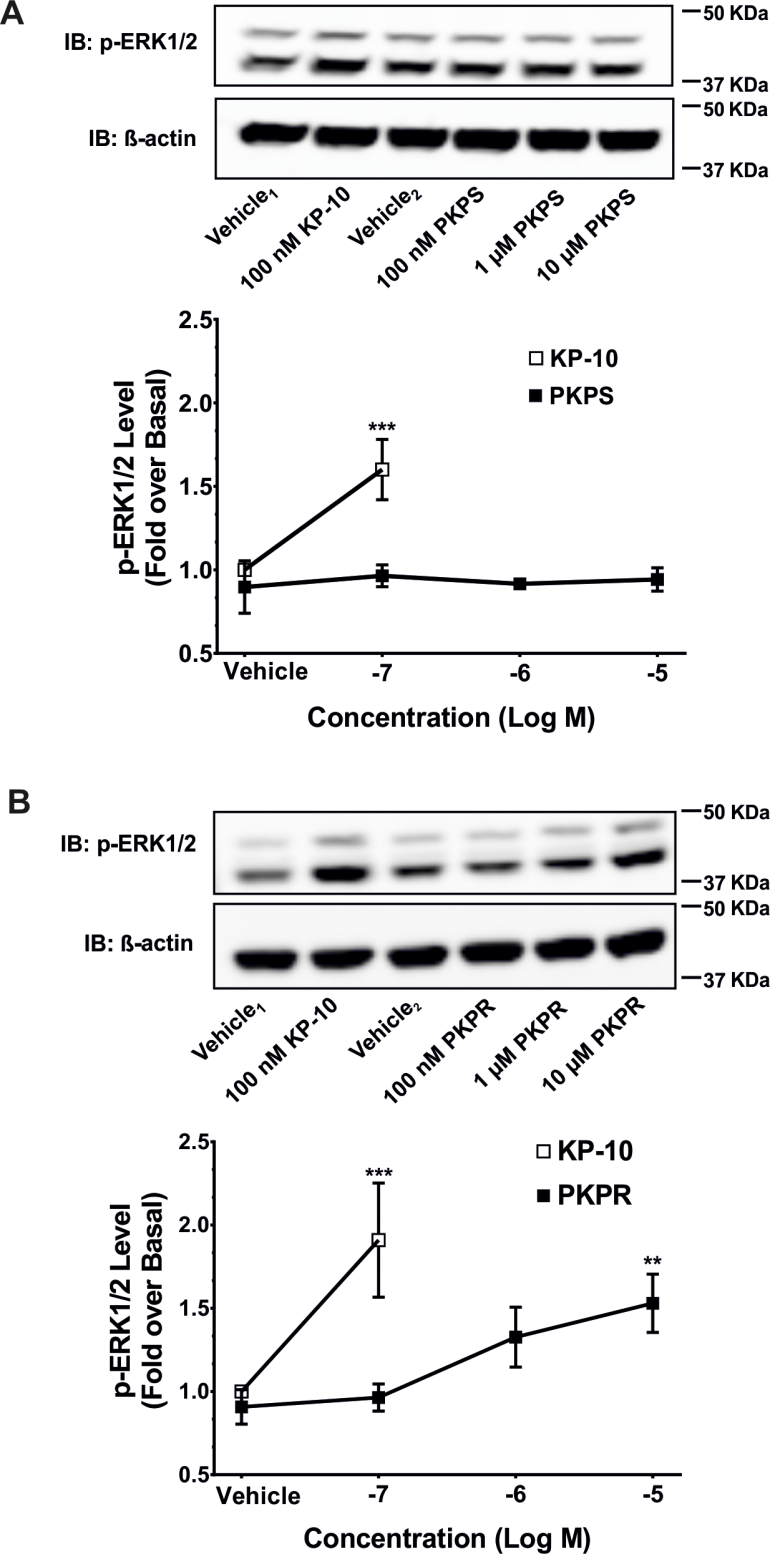
**Stimulation of the phosphorylation of ERK1/2 by KP-10, PKPS (A) or PKPR (B).** HEK293 cells transiently expressing kisspeptin receptor were starved for 20 hours. The cells were then treated with vehicle (vehicle_1_, 0.02% (v/v) propylene glycol or vehicle_2_, 0.02% (v/v) DMSO), 100 nM KP-10 (□) or increasing concentrations of PKPS or PKPR (■) for 3 minutes. Western blot analyses were done using monoclonal anti-phospho-ERK1/2 and anti-β-actin antibodies. Representative western blot and densitometric quantitation are shown. The data represent the mean fold-changes over the basal of the vehicle-treated control ± SD of three independent experiments. **, *P* < 0.01; ***, *P* < 0.005, compared with the basal.

The agonistic activity of the phosphinic peptides on the kisspeptin receptor was evaluated by measuring their stimulation of the dual phosphorylation of ERK1/2 in HEK293 cells transiently expressing kisspeptin receptor. KP-10 at a final concentration of 100 nM was used as a positive control. Three different concentrations of each KP phosphonic peptide were used to stimulate the cells for 3 minutes at which the maximum response was achieved for KP-10 (S1 Fig). Stimulation of HEK293 cells transiently expressing kisspeptin receptor with KP-10 (100 nM) led to a 1.60-fold increase (*P* < 0.005) in the phosphorylation of ERK1/2 (Fig 3), while neither lower concentrations of PKPR (< 1 μM, Fig 3B) nor PKPS up to 10 μM (Fig 3A) induced phosphorylation of ERK1/2. However, PKPR at a concentration of 10 μM increased the level of phosphorylation of ERK1/2 to a 1.53 ± 0.175-fold over basal (*P* < 0.01) (Fig 3B), indicating that PKPR is biologically active.

### Examination of the antagonistic activity of KP phosphinic peptides on kisspeptin receptor

**Fig 4:**
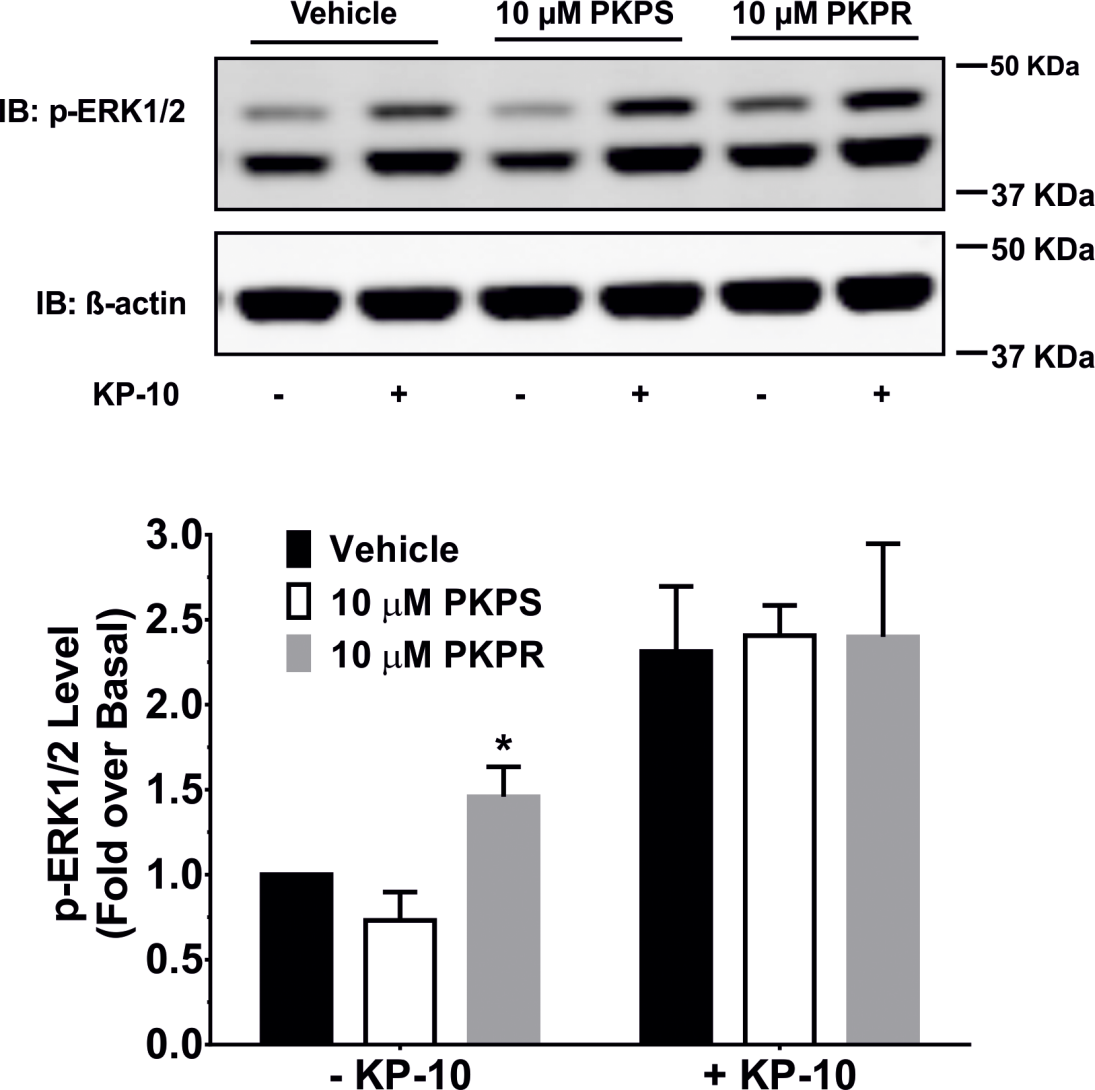
Effect of PKPS and PKPR on KP-10-induced phosphorylation of ERK1/2. MCF-7 cells transiently expressing kisspeptin receptor were serum-starved for 20 hours. The cells were then treated with vehicle (0.02% (v/v) DMSO; black bars), 10 μM PKPS (white bars) or PKPR (grey bars) for 30 minutes. After that, vehicle (0.02% (v/v) propylene glycol, labelled–KP-10) or 100 nM KP-10 (labelled + KP-10) was added for a further 10-minute stimulation. Western blot analyses were carried out using monoclonal anti-phospho-ERK1/2 and anti-β-actin antibodies. Representative western blot (upper) and densitometric quantitation (bottom) are shown. The data represent the mean fold-changes over the basal treated with vehicle ± SD of three independent experiments. *, *P* < 0.05, compared with the basal treated with vehicle.

To examine whether the KP phosphinic peptides exhibit antagonistic activity on the kisspeptin receptor, their ability to inhibit KP-10-induced phosphorylation of ERK1/2 was tested. MCF-7 cells transiently expressing kisspeptin receptor were used as model cells. The cells were first treated with 10 μM of PKPS or PKPR respectively for 30 minutes. After that, KP-10 (100 nM, final concentration) was added for a further 10-minute stimulation which itself elicited a significant (*P* < 0.001) phosphorylation of ERK1/2 (S1 Fig). However, PKPS (Fig 4; white bars) or PKPR (Fig 4; grey bars) at 10 μM did not inhibit KP-10-induced phosphorylation of ERK1/2, while treatment of the cells with PKPR alone, i.e., in the absence of KP-10, gave a 1.46 ± 0.176-fold increase (*P* < 0.05) in the phosphorylation of ERK1/2, similar to the result obtained above.

### Evaluation of the binding of KP phosphinic peptides to kisspeptin receptor

**Fig 5:**
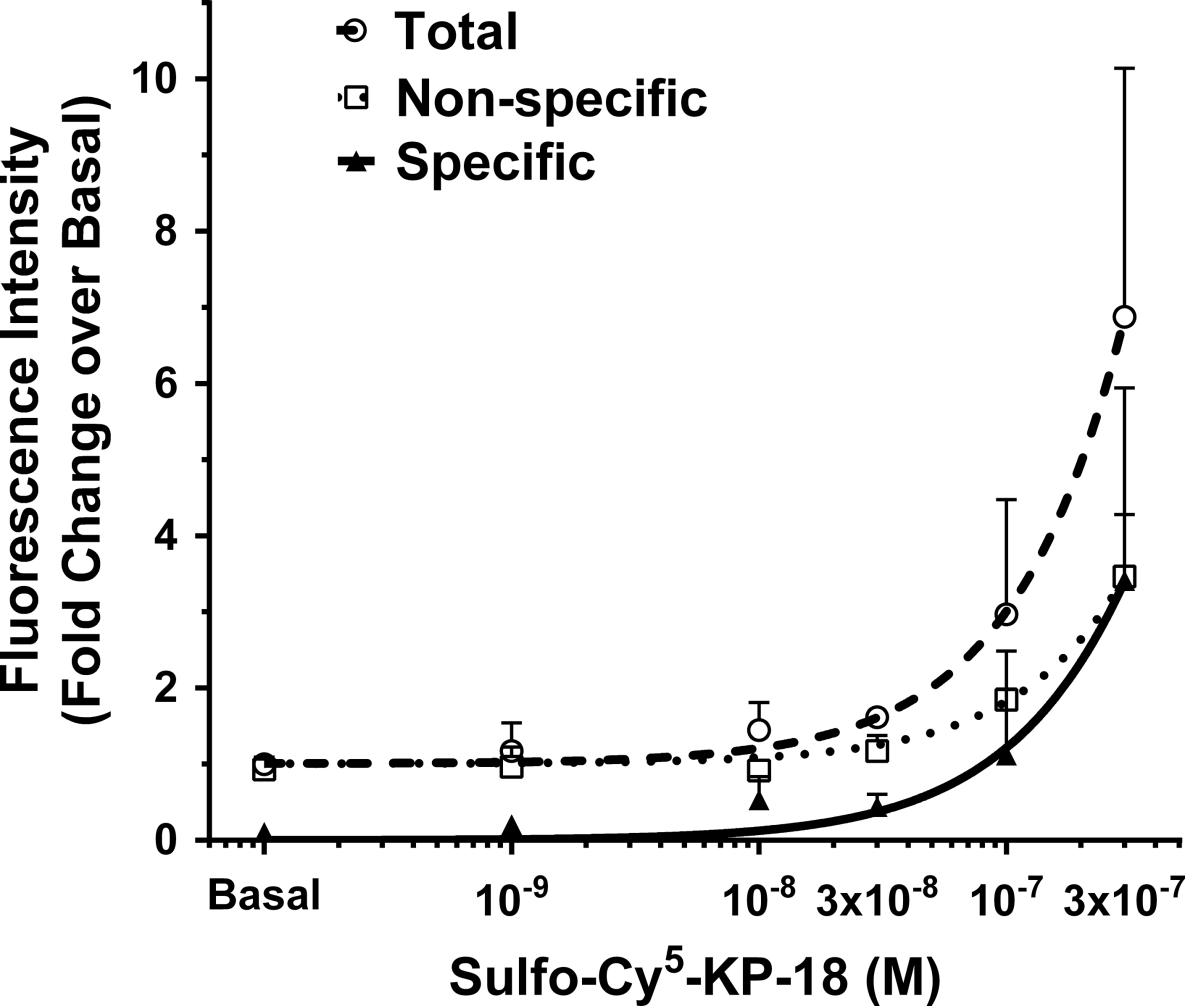
Binding of Sulfo-Cy^5^-KP-18 to kisspeptin receptor. HEK293 cells were transiently transfected with kisspeptin receptor and cultured for 48 hours. The cells were then incubated with increasing concentrations of Sulfo-Cy^5^-KP-18 at 4°C for 4 hours. Total binding of Sulfo-Cy^5^-KP-18 was measured by fluorescence intensity after washing off free ligand. Non-specific binding was determined in the presence of 10 μM KP-10. The mean fold-changes over the basal of the fluorescence intensity ± SD from three independent experiments were calculated and plotted. Specific binding was calculated by subtracting non-specific binding from the total. Total binding (○; dash curve), non-specific binding (□; dot curve) and specific binding (▲; solid curve).

A fluorophore-labelled KP-18 was designed and synthesized in order to determine the binding of PKPS and PKPR to kisspeptin receptor. To minimize the effect of the conjugate of the water-soluble fluorophore Sulfo-Cy^5^ on the function of the C-terminal 10 amino acids of KPs, Sulfo-Cy^5^ was introduced to the N-terminal amine of KP-18, as the introduction of Cy^5.5^ to the N-terminal amine of KP-10 led to inactivity of the Cy^5.5^-KP-10 in binding with kisspeptin receptor [41]. A saturation binding assay was carried out in HEK293 cells transiently expressing kisspeptin receptor to detect the binding of Sulfo-Cy^5^-KP-18 to kisspeptin receptor. A significant specific binding was detected at 50 nM or higher concentrations of Sulfo-Cy^5^-KP-18 (Fig 5).

**Fig 6:**
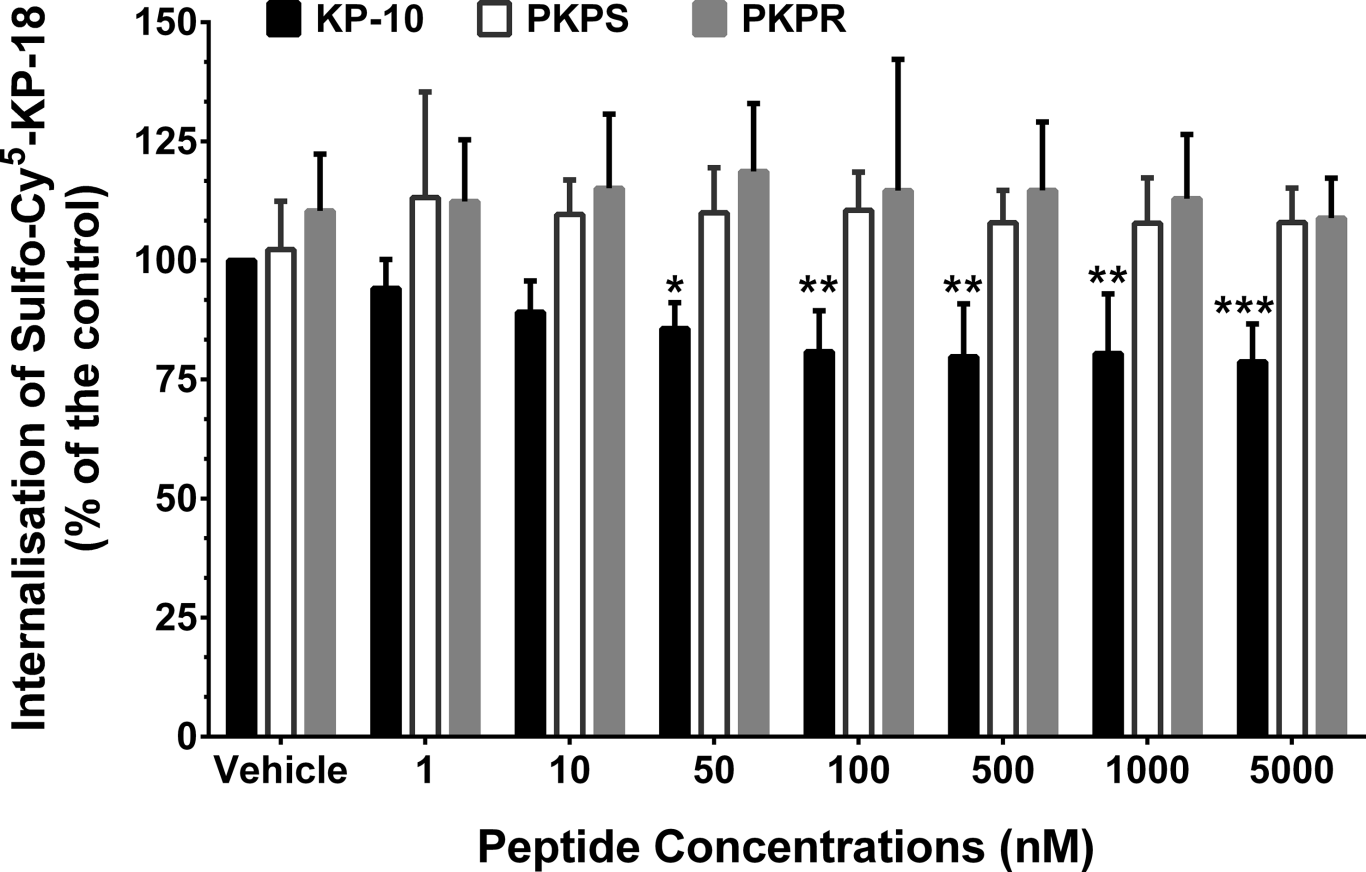
Effect of KP phosphinic peptides on Sulfo-Cy^5^-KP-18-induced internalisation of kisspeptin receptor. MCF-7 cells transiently expressing kisspeptin receptor were co-incubated with 50 nM of Sulfo-Cy^5^-KP-18 and vehicle (control), different concentrations of KP-10 (black bars), PKPS (white bars) or PKPR (grey bars) respectively for 30 minutes. The internalisation of Sulfo-Cy^5^-KP-18 was normalised as percentage of that (control) measured in the absence of KP-10 (vehicle only). The data represent the mean percentage-changes of four independent experiments. *, *P* < 0.05; **, *P* < 0.01; ***, *P* < 0.005, compared with the control.

Since the saturation of Sulfo-Cy^5^-KP-18 binding was not observed even though high concentrations of Sulfo-Cy^5^-KP-18 were used, the dissociation constant, K_d_ value of Sulfo-Cy^5^-KP-18, could not be determined. The lower sensitivity of the Sulfo-Cy^5^-KP-18 binding assay made it impossible to be used as a labelled ligand for competition binding assay to measure the binding affinity of the KP phosphinic peptides. As an alternative approach, the binding of the KP phosphinic peptides to kisspeptin receptor was examined by measuring their inhibition on Sulfo-Cy^5^-KP-18-induced internalisation of the kisspeptin receptor. MCF-7 cells transiently expressing with kisspeptin receptor were co-incubated with 50 nM Sulfo-Cy^5^-KP-18 and vehicle, different concentrations of KP-10, PKPS or PKPR respectively. Addition of KP-10 at 5 μM led to a maximum (22.3% ± 7.96) inhibition (*P* < 0.005) on Sulfo-Cy^5^-KP-18-induced internalisation of the kisspeptin receptor (Fig 6; black bars). However, neither PKPS nor PKPR inhibited the internalisation of Sulfo-Cy^5^-KP-18 at any concentrations tested (Fig 6).

### Inhibition of KP phosphinic peptides on MMPs

**Fig 7:**
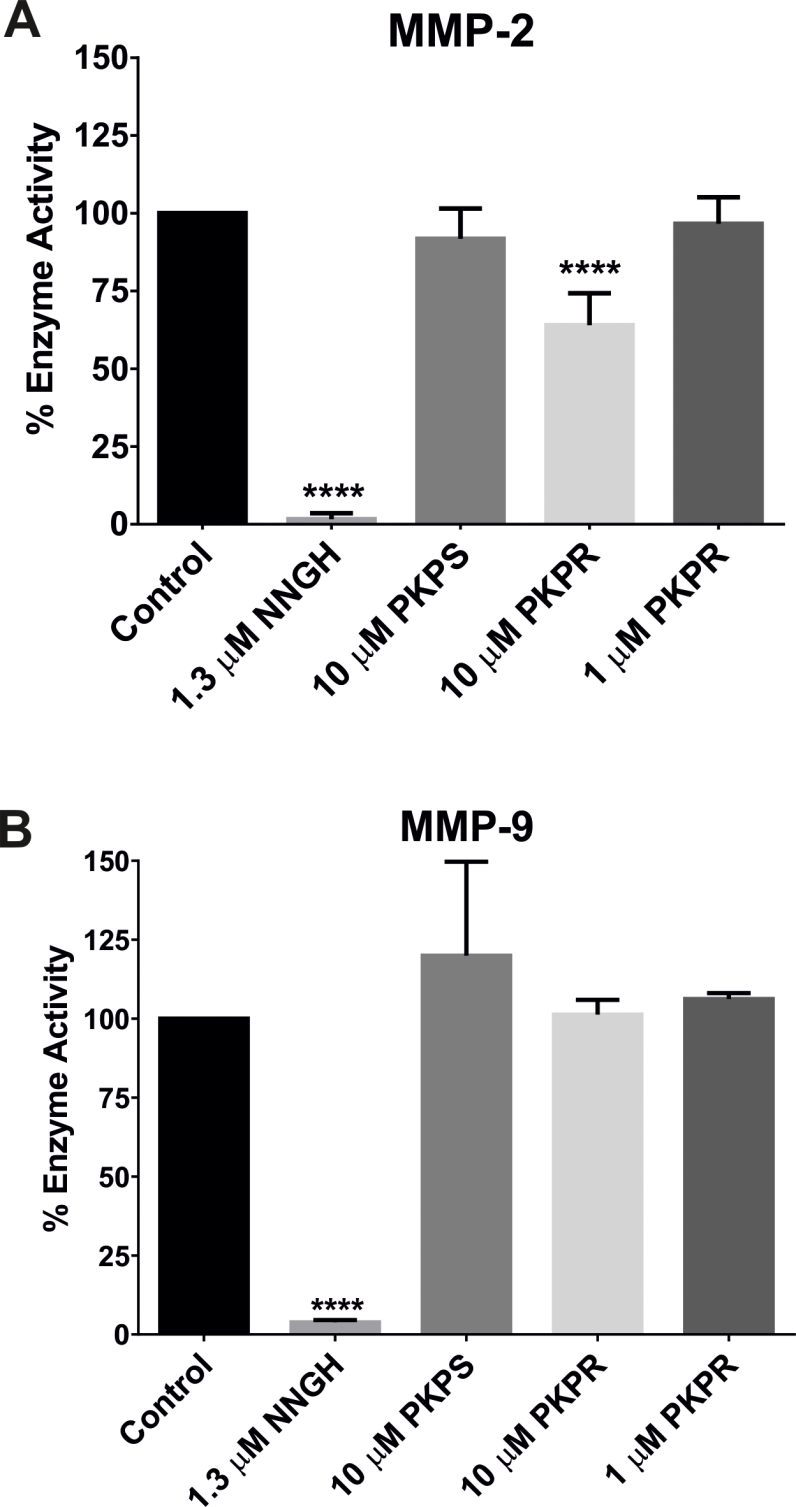
**Effect of KP phosphinic peptides on MMP-2 (A) or MMP-9 (B) enzyme activity.** The inhibition assays on MMPs were conducted using MMP colorimetric drug discovery kits in 96-well microtiter plates as per the manufacturer’s instructions. A final concentration of 10 μM PKPS, 10 μM PKPR, 1 μM PKPR, or 1.3 μM NNGH (positive control) as indicated were respectively added to the assay mixture containing MMP-2 or MMP-9 enzyme. The maximum activity (control) was measured in the absence of inhibitors. The data represent the mean percentage-changes over the control ± SD from three independent experiments. ****, *P* < 0.001 compared with the control.

The effect of the KP phosphinic peptides on MMP-2 and MMP-9 enzyme activity was detected using MMP colorimetric drug discovery kits. NNGH, a potent inhibitor of MMPs, was provided by the kits and used as a positive control. NNGH at 1.3 μM almost completely inhibited the activities of both MMP-2 and MMP-9 (Fig 7). PKPR at a final concentration of 10 μM inhibited the activity of MMP-2 by 35.98% ± 10.22 (*P* <0.001, Fig 7A). However, PKPS and a lower concentration of PKPR did not inhibit the activity of MMP-2. Moreover, none of the KP phosphine peptides inhibited the activity of MMP-9 (Fig 7B).

## Discussion

KPs and their cognate receptor play important roles in the regulation of cancer metastasis and reproduction and, therefore, are important targets for therapeutic intervention. However, natural KPs are cleaved rapidly by various serum-containing proteases especially MMPs, and, therefore, are metabolically unstable [22–24]. In order to explore the potential development of KP analogues with improved metabolic stability, especially with inhibition to MMPs, two KP phosphinic peptides containing a replacement of the peptide bond between Gly^51^-Leu^52^ with a phosphinic acid moiety were synthesized based on the primary sequence of KPs (Figs 1 and 2).

The agonistic activity of the KP phosphinic peptides on the kisspeptin receptor was assessed by measurement of their stimulation on the phosphorylation of ERK1/2. The assay was carried out on HEK293 cells transiently expressing kisspeptin receptor, given that HEK293 cells have high transfection efficiency for cDNA and low basal activity of ERK1/2 phosphorylation. In addition, HEK293 cells were reported to express kisspeptin receptor naturally which regulates embryonic kidney morphogenesis and glomerular development [39], although no kisspeptin receptor binding and activity were detected in our cells. The results showed that PKPR activated kisspeptin receptor at 10 μM (Fig 3B), indicating that it possesses the capability to bind and activate kisspeptin receptor. By contrast, its enantiomer (PKPS) did not stimulate ERK1/2 phosphorylation (Fig 3A), indicating that the pseudo-Leu residue is stereochemically essential for kisspeptin receptor binding and activation. This is consistent with the observation that individual substitution of the last five amino acids at C-terminus of KP-10 with the corresponding D-enantiomer leads to the complete loss of the agonistic activities of the peptides (Niida et al., 2006).

Since the introduction of a dipeptide isostere structure to biologically functional peptides may alter their original bioactivity [32], the kisspeptin receptor-antagonistic activity of PKPS and PKPR was also examined by detecting their inhibition of KP-10-induced phosphorylation of ERK1/2. The results showed that neither PKPS nor PKPR inhibited the KP-10-induced phosphorylation of ERK1/2 (Fig 4). Further examination of the effect of KP phosphinic peptides on the fluorescence-labelled agonist-induced internalization of kisspeptin receptor was carried out using a similar method described by Tomita and colleagues [42]. Introduction of Cy^5.5^ to the N-terminal amine of KP-10 leads to the fluorescence-labelled ligand being functionally inactive [41]. Here, finding that the linkage of a water-soluble Sulfo-Cy^5^ to the N-terminal amine of KP-18 retains the peptide binding capability with kisspeptin receptor (Fig 5). However, a significant receptor binding of the fluorescence-labelled ligand could only be detected at 50 nM or higher concentrations of Sulfo-Cy^5^-KP-18. A similar result was reported for tetramethylrhodamine and rhodamine green-labelled KP-14 or KP-52 [43]. The development of the functional Sulfo-Cy^5^-KP-18 allowed us to determine potential binding of the KP phosphinic peptides via their inhibition to Sulfo-Cy^5^-KP-18-induced receptor internalisation. Co-incubation of KP-10 at concentrations ≥ 50 nM with Sulfo-Cy^5^-KP-18 significantly inhibited the internalisation of Sulfo-Cy^5^-KP-18 with a maximum inhibition of about 22%. However, no inhibition was observed for KP phosphinic peptides, even at a high concentration of 5 μM, indicating that PKPS and PKPR may not bind to kisspeptin receptor or bind to the receptor with a relatively low affinity. In combination with the functional results of KP phosphinic peptide-induced ERK1/2 phosphorylation, we propose that PKPS does not bind to kisspeptin receptor, while PKPR binds with kisspeptin receptor at a relatively low affinity, as stimulation of HEK293 cells transiently expressing kisspeptin receptor by 10 μM PKPR leads to phosphorylation of ERK1/2.

In order to check if introduction of a non-hydrolysable ~[P(O)(OH)CH2]~ group at the cleavage site of KPs leads to inhibition of the peptides to zinc metalloproteases, their ability to inhibit the activity of MMP-2 and MMP-9 was further evaluated. A significant inhibition of PKPR at 10 μM on MMP-2 enzyme activity was observed (Fig 7A) while PKPS had no effect on MMP-2 activity, indicating the specificity of PKPR to MMP-2. By contrast, both PKPS and PKPR did not inhibit the activity of MMP-9 (Fig 7B), indicating that PKPR is a selective inhibitor toward MMP-2. In addition, the result also indicates that PKPR may form a stable complex with MMP-2 and, thus, may be resistant to MMP-2-mediated hydrolysis. Currently, most inhibitors of zinc-metalloproteases, such as MMPs, are designed based on the usage of a strong zinc-chelating group, such as carboxylate and hydroxamic groups [34]. Although these peptides provide high potency, they exhibit low selectivity, indicating that their inhibition may mainly rely on the interaction between the zinc-chelating group and the zinc ion. In contrast, the phosphoryl group of phosphinic peptides is a weak zinc-chelating group and, as a consequence, only the peptides that allow optimal and sufficient intermolecular interactions with the enzyme active sites will provide inhibition. Moreover, phosphinic peptides mimic the structure of the substrate in the transition state [33, 34]. Thus, phosphinic peptides may provide an opportunity to develop potent and selective inhibitors for zinc-metalloproteases which are involved in promoting cancer metastasis. PKPR presented here may, hence, serve as a lead compound for further modification to develop a potent and selective inhibitor of MMP-2.

In conclusion, our studies propose a new option to develop novel KP phosphinic analogues, such as PKPR, which activate kisspeptin receptor and inhibit the activity of MMPs, by replacing the peptide bond between Gly^51^ and Leu^52^ with a phosphinic acid moiety. PKPR may serve as a lead for further development of a metabolically stable peptide analogue for hormone replacement therapies and/or of a potent anti-metastasis agent targeting both kisspeptin receptor and MMPs.

## Supporting information

S1 Fig **Time-course of the phosphorylation of ERK1/2 induced by KP-10 in HEK293 (A) and MCF-7 (B) cells.** Cells transiently expressing kisspeptin receptor were starved for 20 hours. The cells were then treated with vehicle (0.02% (v/v) propylene glycol) or 100 nM KP-10 for different times as indicated. Western blot analyses were carried out using monoclonal anti-phospho-ERK1/2 and anti-β-actin antibodies. Representative western blot and densitometric analysis are shown. The data represent the mean fold-change over basal (vehicle treated control) ± SD of three independent experiments. **, *P* < 0.01; ****, *P* < 0.001, compared with the basal.
(TIF)

S2 Fig Entire blots of the representative western blots plotted in Fig 3. HEK293 cells expressing kisspeptin receptor were stimulated by PKPS (A) or PKPR (B) and the phosphorylation of ERK1/2 was measured by western blotting.
(TIF)

S3 Fig Entire blots of the representative western blots plotted in Fig 4. Effect of PKPS and PKPR on KP-10-induced phosphorylation of ERK1/2.
(TIF)

S4 Fig Entire blots of the representative western blots plotted in S1 Fig. Phosphorylation of ERK1/2 stimulated by KP-10 in HEK293 (A) and MCF-7 (B) cells.
(TIF)
