# Supplementary figures and images for: Functional examination of novel kisspeptin phosphinic peptides
Source: PLoS One. 2018 Apr 3;13(4):e0195089. doi: 10.1371/journal.pone.0195089 (PMC5882139; doi:10.1371/journal.pone.0195089)

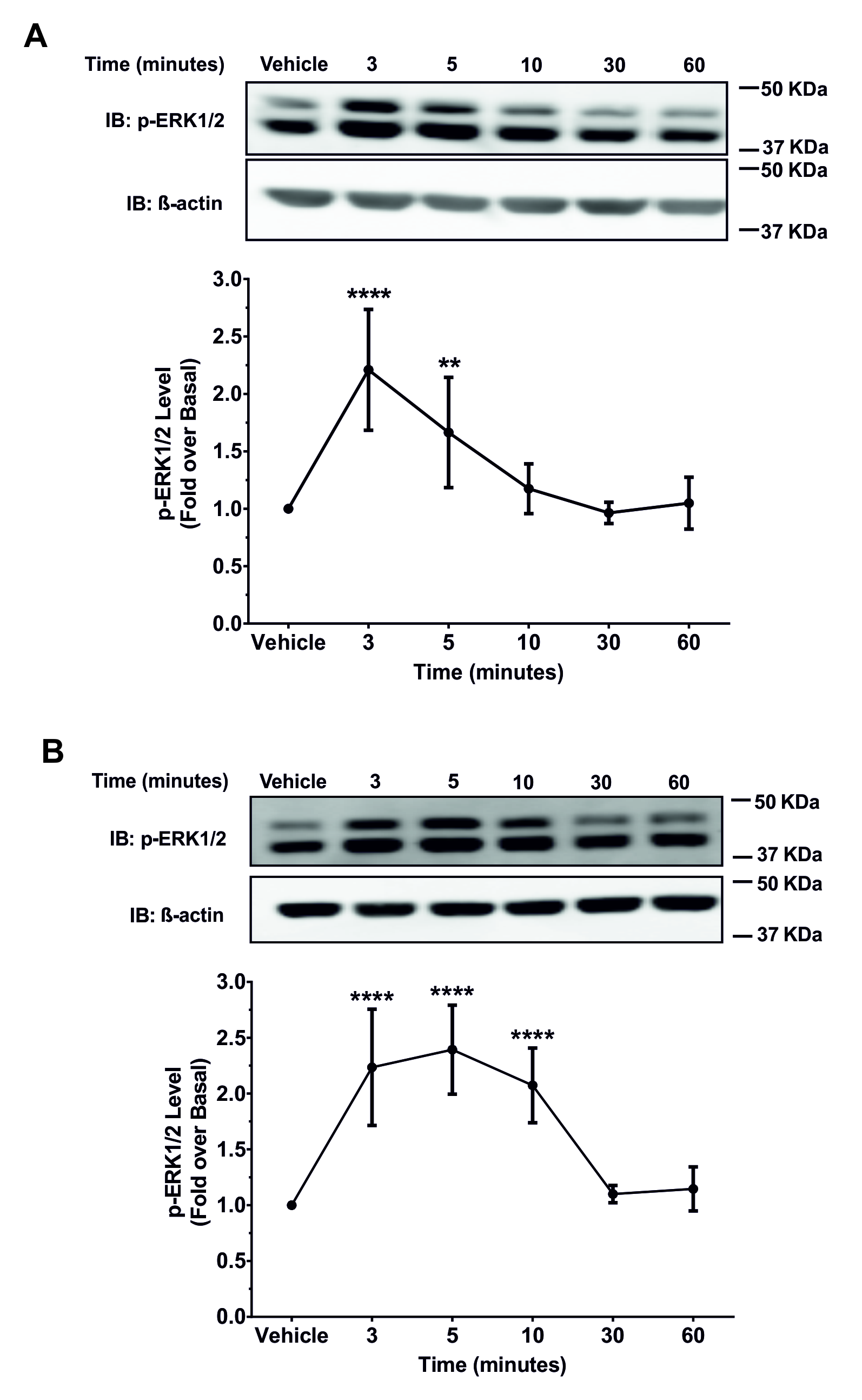

Supplement: S1 Fig — Time-course of the phosphorylation of ERK1/2 induced by KP-10 in HEK293 (A) and MCF-7 (B) cells. Cells transiently expressing kisspeptin receptor were starved for 20 hours. The cells were then treated with vehicle (0.02% (v/v) propylene glycol) or 100 nM KP-10 for different times as indicated. Western blot analyses were carried out using monoclonal anti-phospho-ERK1/2 and anti-β-actin antibodies. Representative western blot and densitometric analysis are shown. The data represent the mean fold-change over basal (vehicle treated control) ± SD of three independent experiments. **, P < 0.01; ****, P < 0.001, compared with the basal. (TIF) [file pone.0195089.s001.tif]

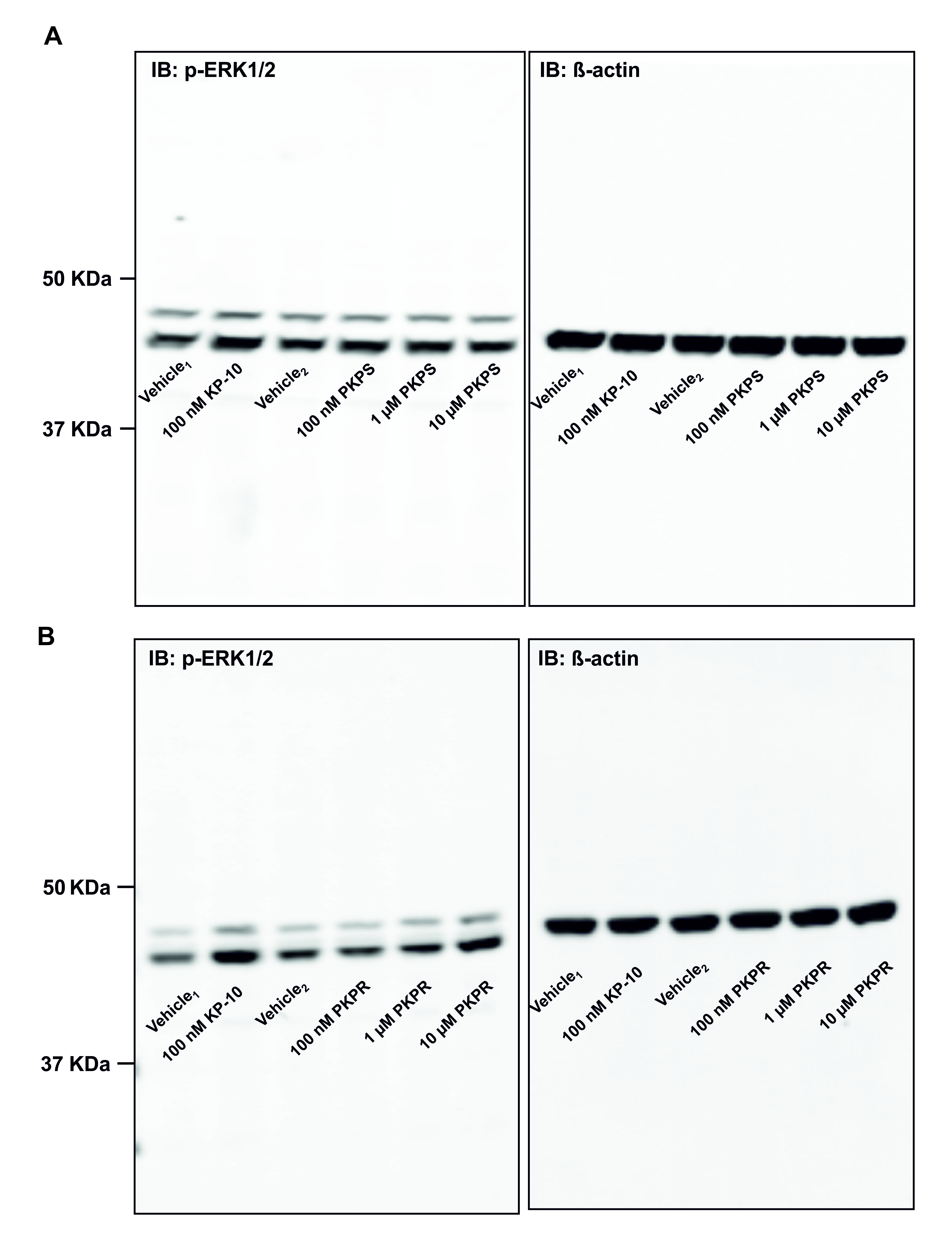

Supplement: S2 Fig — HEK293 cells expressing kisspeptin receptor were stimulated by PKPS (A) or PKPR (B) and the phosphorylation of ERK1/2 was measured by western blotting. (TIF) [file pone.0195089.s002.tif]

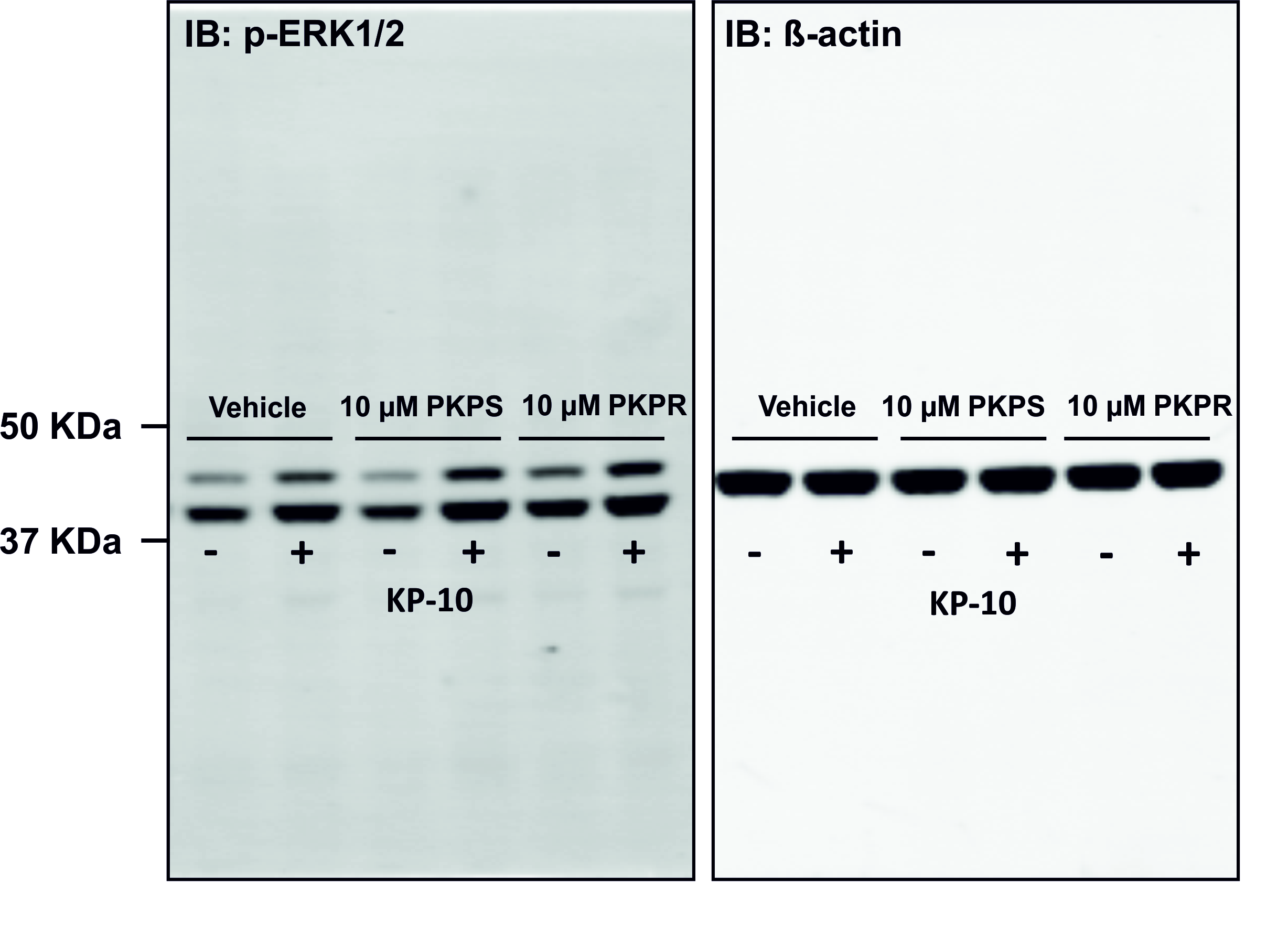

Supplement: S3 Fig — Effect of PKPS and PKPR on KP-10-induced phosphorylation of ERK1/2. (TIF) [file pone.0195089.s003.tif]

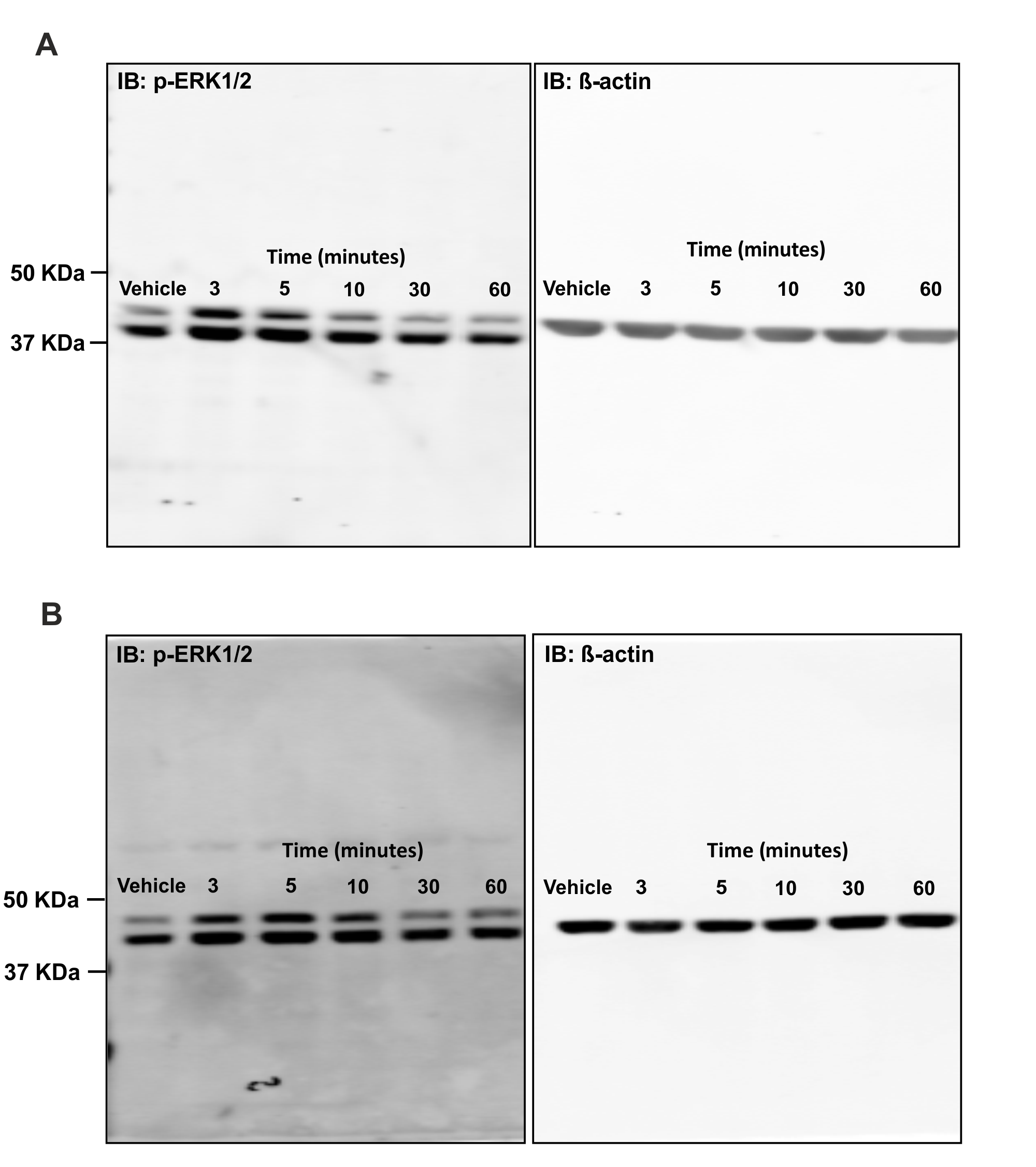

Supplement: S4 Fig — Phosphorylation of ERK1/2 stimulated by KP-10 in HEK293 (A) and MCF-7 (B) cells. (TIF) [file pone.0195089.s004.tif]
